# Root-localized aortic dissection in Loeys–Dietz syndrome diagnosed with the coronary computed tomography angiography: a case report

**DOI:** 10.1093/ehjcr/ytag178

**Published:** 2026-03-10

**Authors:** Ikumi Inoue, Shunsuke Inoguchi, Yasushi Ino, Keizo Kimura, Atsushi Tanaka

**Affiliations:** Department of Internal Medicine, Kinan Hospital, 46-70, Shinjo-cho, Tanabe, Wakayama 646-8588, Japan; Department of Cardiology, Kinan Hospital, 46-70, Shinjo-cho, Tanabe, Wakayama 646-8588, Japan; Department of Cardiology, Kinan Hospital, 46-70, Shinjo-cho, Tanabe, Wakayama 646-8588, Japan; Department of Cardiology, Kinan Hospital, 46-70, Shinjo-cho, Tanabe, Wakayama 646-8588, Japan; Department of Cardiology, Kinan Hospital, 46-70, Shinjo-cho, Tanabe, Wakayama 646-8588, Japan; Department of Cardiovascular Medicine, Wakayama Medical University, 811-1 Kimiidera, Wakayama, Wakayama 641-8509, Japan

**Keywords:** Case report, Type A aortic dissection, Root-localized aortic dissection, Loeys-Dietz syndrome, Cardiac-gated CT

## Abstract

**Background:**

Aortic dissection is caused by a tear in the inner layer of the aorta, and blood comes between the layers of the aortic wall. Contrast-enhanced computed tomography (CT) is typically considered to be useful in its diagnosis. However, in cases of aortic dissection localized to the aortic root, diagnosis with CT or echocardiography can be challenging.

**Case Summary:**

We report the case of a patient with root-localized aortic dissection that presented with acute aortic valve regurgitation. Contrast-enhanced chest CT revealed marked dilation of the aortic root, but it did not show evidence of dissection. However, there was a marked dilation of the aortic root and acute aortic regurgitation, which suggested root-localized dissection. Conventional CT was limited by motion artefacts and did not demonstrate dissection, so we decided to perform cardiac-gated CT to achieve a more accurate evaluation of the aortic root. This allowed us to make a definite diagnosis and proceed to life-saving surgery.

**Discussion:**

Acute type A-localized aortic dissection has several clinical features that were distinct from acute type A aortic dissection, which we summarize with prior cases. We found cardiac-gated CT to be useful in making definite diagnosis in this case.

Learning points

- Root-localized aortic dissection may lead to delayed diagnosis.
- Cardiac-gated CT may be useful in the diagnosis in such cases.
- Root-localized aortic dissection may occur in relatively young patients when associated with hereditary diseases, such as Loeys–Dietz syndrome.

## Introduction

Aortic dissection is caused by a tear in the inner layer of the aorta and the escaping blood causes a split between the layers of the aortic wall. Stanford type A aortic dissection (ATAAD) is defined as a dissection involving the ascending aorta, regardless of the site of the primary intimal tear, and is a life-threatening condition requiring urgent diagnosis and surgical intervention. Approximately 85% of cases of ATAAD present with chest or back pain, but others are asymptomatic.^1^ Transthoracic and transoesophageal echocardiography and contrast-enhanced computed tomography (CT) are both considered to be useful for diagnosis.^1^ However, in some cases, the diagnosis can only be made intraoperatively. Here, we report a case in which cardiac-gated contrast-enhanced CT was very useful in the diagnosis of a patient with root-localized aortic dissection (RLAD).

## Summary figure

**Figure:**
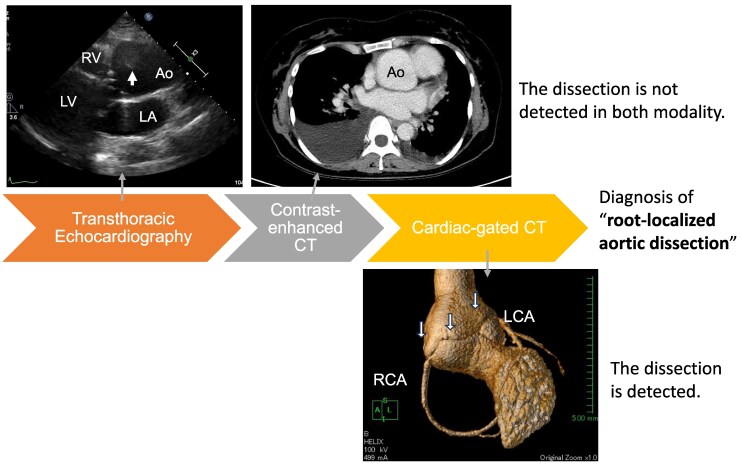


## Case presentation

A 44-year-old woman presented to our hospital with the chief complaint of dyspnoea. She had no notable past medical or family history and was not on any regular medication. However, for approximately 2 weeks prior to admission, she had a productive cough. Orthopnoea developed 1 day before admission, and she was referred to the Department of Cardiology. She denied chest pain and back pain.

**Figure 1:**
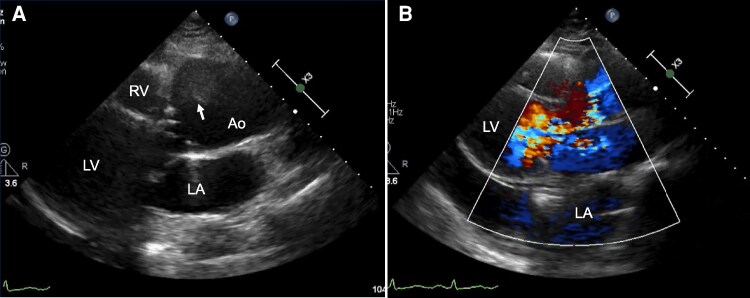
Transthoracic echocardiography findings. (*A*) Enlargement of sulcus of Valsalva, 50 mm, and movable linear structure suspected dissected intimal flap (arrow). (*B*) Severe aortic regurgitation was seen on the parasternal long-axis view. Ao, aorta; LA, left atrium; LV, left ventricle; RA, right atrium; RV, right ventricle.

**Figure 2:**
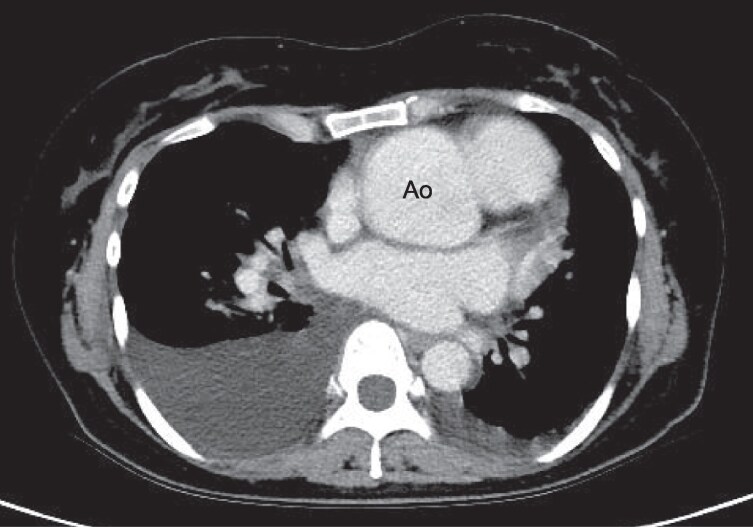
Contrast-enhanced chest computed tomography. Computed tomography showed bilateral pleural effusion and marked dilation of the aortic root (51 mm), but there was no evidence of aortic dissection.

**Figure 3:**
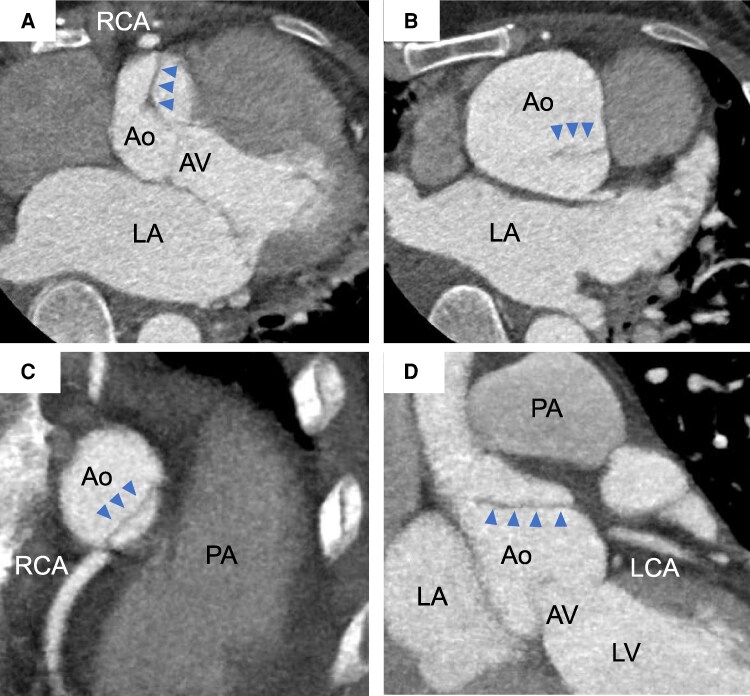
Cardiac-gated computed tomography. Cardiac-gated computed tomography showed aortic dissection (arrowhead) on the ascending aorta in axial view (*A* and *B*), which was extending to near the ostia of both the right and left coronary arteries in sagittal view (*C* and *D*). Ao, aorta; AV, aortic valve; LA, left atrium; LCA, left coronary artery; LV, left ventricle; PA, pulmonary artery; RA, right atrium; RCA, right coronary artery; RV, right ventricle.

**Figure 4:**
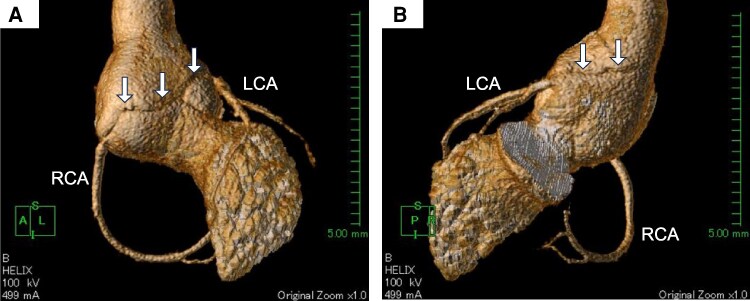
Cardiac-gated 3D computed tomography. 3D reconstruction computed tomography images showed a spiral dissection (arrow) extending from the right coronary ostium to just above the left coronary ostium and reaching the vicinity of the sinotubular junction (*A* and *B*). LCA, left coronary artery; RCA, right coronary artery.

On arrival, her vital signs were temperature 37.7°C, blood pressure 123/54 mmHg, pulse rate 103/min, respiratory rate 28/min, and SpO_2_ 92% on room air. She was 165 cm tall, weighed 62.4 kg (body mass index & 22.9 kg/m^2^), and had no history of smoking. Auscultation revealed a diastolic murmur (Levine grade II/VI) at the left third intercostal space and bilateral wet rales in the lower lungs. No specific musculoskeletal abnormalities were observed. Notable results of laboratory analysis included D-dimer of 3.60 μg/dL (0–1.0 μg/dL) and brain natriuretic peptide of 611.1 pg/mL (≤20 pg/mL). The patient did not have dyslipidaemia or diabetes. Transthoracic echocardiography showed significant dilation of the right coronary sinus (50 mm) with a flap-like structure and severe aortic regurgitation (*Figure 1*). The findings were insufficient to fully assess the extent of the dissection. Additional imaging with contrast-enhanced chest CT was therefore required to obtain definitive anatomical information for appropriate surgical planning. It revealed marked dilation of the aortic root, but there was no clear evidence of aortic dissection (*Figure 2*). However, we could not yet definitively exclude dissection of the aortic root; therefore, cardiac-gated CT was performed. It revealed flaps near the ostia of both the right and left coronary arteries (*Figure 3*). A horizontally oriented dissection was not clearly identified on axial imaging, so we focused on the 3D reconstructed image, which showed a spiral dissection extending from the right coronary ostium to just above the left coronary ostium and reaching the vicinity of the sinotubular junction (*Figure 4*).

Based on these findings, the patient was diagnosed with acute aortic regurgitation caused by RLAD. She underwent urgent open surgery, during which the ascending aortic root was seen to be significantly dilated, with circumferential dissection. During the Bentall procedure, the dissection was observed in both the inner and outer layers of the right and left coronary arteries. A 26 mm synthetic aortic graft and a mechanical valve were used to construct the composite graft. The coronary buttons were trimmed and anastomosed, ensuring alignment of the dissected inner and outer layers at the sight of the trimming. The post-operative course was stable; however, prolonged hospitalization was required for rehabilitation and optimization of medical therapy. The patient was discharged on post-operative Day 25.

**Figure 5:**
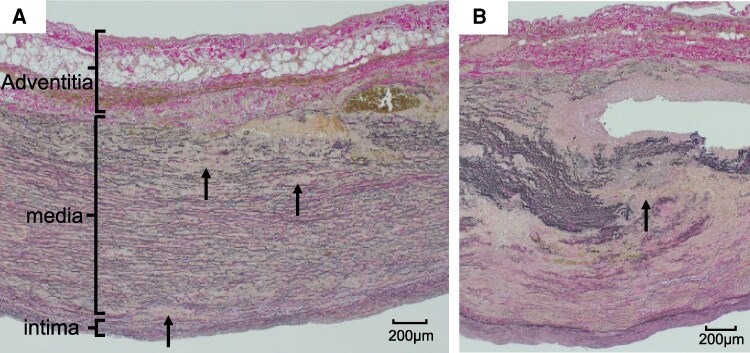
Histological examination of tissue obtained from the sinus of Valsalva. Histological images showed reduced elastic fibres (arrow) in the non-dissected regions (*A*) and extensive loss of elastic fibres and medial rupture caused by rupture of elastic fibre (arrow) in the dissected areas (*B*).

Histological examination of tissue in the non-dissected regions obtained from the sinus of Valsalva revealed scattered areas of reduced elastic fibres, while that in the dissected regions revealed extensive loss of elastic fibres and medial rupture (*Figure 5*). Genetic testing was requested due to the absence of obvious underlying conditions and the relatively young age for a patient with aortic dissection. It revealed a pathogenic mutation in SMAD3, consistent with Loeys–Dietz syndrome (LDS). After the diagnosis of LDS, a detailed physical examination was performed, revealing no evident craniofacial abnormalities such as hypertelorism, nor skeletal features including scoliosis. Given the underlying genetic disorder, regular outpatient follow-up with CT imaging was planned. She has shown a favourable clinical course without recurrence. Furthermore, the patient was informed that family members might harbour the same genetic mutation and that genetic testing was recommended. All clinical management and treatment of the patient were conducted in accordance with the ethical principles outlined in the Declaration of Helsinki, and written informed consent was obtained from the patient.

## Discussion

Root-localized aortic dissection is an exceptionally rare entity. A review of the existing literature identified only seven cases with dissection that was confined to the aortic root. The prevalence of this condition has not been clearly established because of its rarity, and diagnosis is thought to be challenging using only conventional imaging modalities. There seem to be several clinical differences from typical ATAAD. We compared our patient’s case against seven cases of RLAD from the literature (*Table 1*) and reports of patients with ATAAD.^2–7^ The average age of onset was younger in patients with RLAD (54.0 years) than in patients with ATAAD (61.5 years).^8^ Additionally, while 85% of patients with ATAAD reported chest or back pain, only 62.5% of patients with RLAD exhibited this symptom.^1^ In other words, RLAD can present without typical symptoms. The aortic dissection detection risk score was ‘1’ in this case, which was solely based on the presence of a cardiac murmur. Despite the relatively low score, we could not exclude aortic dissection, which highlights the diagnostic challenge of root-localized dissection and the importance of appropriate imaging.

**Table 1:** Clinical description of seven cases of root-localized aortic dissection reported previously and our case

	Age	Sex	Chief symptom	Aortic valve regurgitation	Coronary malperfusion	Diagnostic method
1^2)^	62	F	Chest pain	severe	–	Pre-operative TEE
2^3)^	48	M	Chest pain	Not described	LCA	Catheter intervention
3^4)^	56	M	Chest pain	moderate	RCA	Surgery
4^5)^	48	F	Back pain	Not described	–	TEE
5^6)^	51	M	Back pain	severe	–	TEE
6^6)^	71	M	Dyspnoea	severe	–	TEE
7^7)^	52	M	Dyspnoea	severe	–	TEE
Our case	44	F	Dyspnoea	severe	LCA, RCA	CCTA

AR, aortic valve regurgitation; CCTA, coronary computed tomography angiography; TEE, transoesophageal echocardiography.

Similar to ATAAD, various complications are associated with RLAD. Aortic regurgitation is a notable major complication, which occurred in six of the nine patients in the literature with RLAD, whereas it reportedly occurred in 35.9% (167/524) of patients that underwent repair for ATAAD.^9^

Coronary malperfusion is another major complication warranting mention. The incidence of coronary malperfusion in cases of RLAD in the literature was 37.5%, whereas it is reported to be only 6% in cases of ATAAD.^10^ ATAAD tends to propagate longitudinally along the aorta, but RLAD often expands circumferentially. Among the seven patients with well-documented RLAD patterns, six affected one or two cusps, and only in our patient’s case was there a complete circumferential dissection affecting all cusps, which is suggestive of its rarity. Complications such as aortic regurgitation and coronary malperfusion are thought to occur at a high frequency.

In our case, we could not definitively identify dissection by contrast-enhanced CT. Ultimately, however, cardiac-gated CT facilitated definitive diagnosis, and the patient could be successfully treated with emergency surgery. Five out of the eight reviewed cases were diagnosed using transoesophageal echocardiogram for aortic regurgitation evaluation or pre-operative assessment for root and/or coronary reconstruction. Transoesophageal echocardiogram is suggested to be useful for diagnosis, but it may not always be feasible, depending on the patient’s general condition. Although CT with or without contrast medium is relatively non-invasive and it allows rapid evaluation of the entire aorta, we suggest that motion artefacts due to cardiac activity might sometimes hinder detailed assessment of the root. In particular, aortic wall motion during the cardiac cycle creates curvilinear artefacts in the proximal ascending aorta, especially the aortic root.^11^ Distinguishing between genuine dissection of liner structures from motion artefacts is therefore thought to be difficult. Moreover, in cases like ours, where the dissection is localized and circumferential, CT diagnosis may be even more challenging. Contrast-enhanced CT did not lead to definite diagnosis in three of four cases. However, even when dissection is not visible on contrast-enhanced CT, cardiac-gated imaging may improve diagnostic accuracy, especially in cases of RLAD. Cardiac-gated CT provided us with comprehensive anatomical information, allowing simultaneous assessment of the aortic root, coronary arteries, and potential surgical anatomy. This may be especially relevant in cases with atypical presentation or when concomitant coronary involvement cannot be excluded. Although contrast-enhanced CT remains the gold standard for the diagnosis of acute aortic syndromes, there should also be consideration of the role of cardiac CT angiography as an initial diagnostic modality. With increasing availability of modern CT scanners and standardized protocols, cardiac CT angiography may be feasible even in peripheral centres, and it could contribute to rapid decision-making in selected patients.

Despite the absence of acquired diseases that possibly cause medial degeneration such as hypertension, histopathological examination in our case revealed a marked decrease in elastic fibres within the tunica media, which raises the possibility of a genetic disorder without apparent skeletal muscle abnormalities. Genetic testing revealed a mutation that was consistent with LDS. Loeys–Dietz syndrome Type 3, which is caused by SMAD3 gene mutations, is rare and is characterized by early-onset osteoarthritis and osteochondritis dissecans, often leading to medical consultation.^12^ Aortic aneurysms and cerebral arterial tortuosity are common, and aortic dissections occur in up to 33% of cases.^12^ In a study with 10-year follow-up, the aortic diameter tended to increase by 0.4 mm/year, with 50% of cases requiring elective root replacement.^12^ Loeys–Dietz syndrome therefore predisposes patients to aortic aneurysm and aortic dissection. AAD in patients with LDS has been reported,^13,14^ but the dissection always extended beyond the aortic root into the ascending aorta. To the best of our knowledge, no prior reports have described an LDS-associated aortic dissection that is entirely confined to the aortic root. This case therefore highlights a previously unreported anatomical presentation and underscores the diagnostic difficulty of root-localized dissections in patients with a heritable aortopathy.

## Conclusion

We reported a case of RLAD in a patient with LDS. Contrast-enhanced chest CT revealed marked dilation of the aortic root but no evident dissection. However, cardiac-gated CT revealed flaps near the ostia of both coronary arteries, and the patient could be successfully treated with emergency surgery. Cardiac-gated contrast-enhanced CT may improve diagnostic accuracy in cases of RLAD.

## Data Availability

Data from previously published studies were obtained from the literature and are cited accordingly. Data related to the present case are not publicly available due to patient confidentiality and ethical restrictions.

## Lead author biography

Ikumi Inoue is a senior resident in the Department of Cardiology, Wakayama Medical University, Japan, currently working at Kinan Hospital. Her clinical interests focus on cardiovascular imaging, particularly echocardiography and diagnostic evaluation of heart and aortic diseases. She is undergoing advanced clinical training in cardiology and internal medicine.

## Notes

**Consent:** The authors confirm that written informed consent was obtained from the patient for the submission and publication of this case report, in accordance with COPE guidelines.
